# High-quality draft genome assembly and functional annotation of *Musa textilis* cv. Inosa

**DOI:** 10.3389/fpls.2026.1866360

**Published:** 2026-07-07

**Authors:** Roneil Christian S. Alonday, Julianne Vilela, Damsel C. Bangcal-Villariño, John Ivan I. Pasquil, Kaito O. Furusho, Adrian Sam Arcillo, Maiah Cheng, Maria Genaleen Q. Diaz, Antonio G. Lalusin, Antonio C. Laurena

**Affiliations:** 1 Philippine Genome Center Program for Agriculture, Livestock, Fisheries, and Forestry, Office of the Vice Chancellor for Research and Extension, University of the Philippines Los Baños, College, Laguna, Philippines; 2 Institute of Plant Breeding, College of Agriculture and Food Science, University of the Philippines Los Baños, College, Laguna, Philippines; 3 The Roslin Institute, The University of Edinburgh, Midlothian, United Kingdom; 4 Institute of Crop Science, College of Agriculture and Food Science, University of the Philippines Los Baños, College, Laguna, Philippines; 5 Institute of Biological Sciences, College of Arts and Sciences, University of the Philippines Los Baños, College, Laguna, Philippines

**Keywords:** fiber quality, genome annotation, genome assembly, *Musa textilis*, PacBio HiFi, Inosa

## Abstract

**Introduction:**

Abaca (*Musa textilis* Née) is an important fiber crop cultivated primarily in the Philippines and valued for its exceptional fiber strength and industrial applications. Despite its economic importance, genomic resources for abaca remain limited, constraining efforts in molecular breeding and trait improvement. Here, we present a high-quality de novo genome assembly and functional annotation of *M. textilis* cv. Inosa, a commercially important cultivar known for superior fiber quality.

**Methods:**

The genome was sequenced using PacBio HiFi technology and assembled de novo, followed by repeat annotation, gene prediction, functional characterization, and comparative genomic analyses with other *Musa* genomes. Orthology, synteny, and fiber-related gene analyses were performed to investigate genome evolution and identify genes associated with fiber development.

**Results:**

The assembled genome spans 612.5 Mb across 388 contigs, with a contig N50 of 9.02 Mb and a BUSCO completeness score of 98.9%, indicating high assembly quality and completeness. Functional annotation identified 37,403 high-confidence protein-coding genes. Repetitive elements account for 59.18% of the genome, representing one of the highest repeat contents reported among *Musa* genomes. Notably, Polinton transposons, a rarely reported transposable element class in *Musa*, were identified. Comparative genomic analyses revealed strong macrosyntenic conservation with other *M. textilis* assemblies and identified 226 Inosa-specific orthogroups. In addition, 348 proteins associated with fiber biosynthesis were annotated, including key enzymes and regulatory proteins involved in cellulose and lignin biosynthesis pathways.

**Discussion:**

This high-quality genome assembly expands the genomic resources available for abaca and provides insights into genome organization, repeat landscape, and fiber-related gene content. The genome will support comparative genomics, marker development, and breeding strategies aimed at improving fiber quality, disease resistance, and climate resilience in abaca.

## 1 Introduction

Natural fiber crops such as *Musa textilis* (abaca), *Gossypium hirsutum* (cotton), and *Corchorus capsularis* (jute) are critical components of sustainable industries due to their biodegradability, strength, and environmental advantages (Chaudhry, 2010; Drieling et al., 2010; Göltenboth and Mühlbauer, 2010; Rahman, 2010). Among these, abaca fibers stand out for their high tensile strength, saltwater resistance, and flexibility (Uppal et al., 2022). Despite its economic significance, especially in the Philippines, where it is endemic, genomic resources for abaca are still limited compared to cotton and jute (Islam et al., 2017; Zhang et al., 2021; Li et al., 2021; Singh and Sarkar, 2022; Yang et al., 2022; Afzal et al., 2022; Khan et al., 2022; Wen et al., 2023; Li et al., 2024).

Abaca (*Musa textilis* L. Nee) is an endemic species of the Philippines primarily cultivated for its strong and durable fibers derived from its leaf sheaths. These fibers, known as Manila hemp, are among the strongest natural fibers and are renowned for their resistance to saltwater, mechanical strength, flexibility, and durability. These properties underpin their extensive industrial applications, particularly in the production of ropes used in ships, twines, fabrics, and specialty papers (Unal et al., 2020; Moreno and Protacio, 2012). The Philippines harbors a diverse collection of abaca cultivars—over 40 identified varieties—yet only three are currently registered under the National Seed Industry Council (NSIC): Abuab, Inosa, and Tangongon (Yllano et al., 2020; Viscarra et al., 2017). Of the three, only Abuab has a whole genome assembly and annotation. However, the existing Abuab assembly remains fragmented (N50 ~47 kb), limiting downstream analyses (Galvez et al., 2021). This limitation highlights the need for additional high-quality assemblies to fully leverage abaca’s potential, underscoring the imperative to sequence other varieties, such as Inosa, to aid comparative genomics and accelerate crop improvement.

From a scientific standpoint, high-quality genome assemblies provide essential foundations for understanding genome evolution, identifying genes linked to complex traits, and constructing a pan-genome for the species (Jung et al., 2019; Bayer et al., 2020; Kong et al., 2023). Such genomic resources enable downstream applications, including marker-assisted selection, genome-wide association studies (GWAS), and CRISPR-based functional validation (Varsheney et al., 2005; Feng et al., 2013).

Here, we present a high-quality genome assembly and functional annotation of *M. textilis* cv. Inosa using PacBio HiFi sequencing technology and advanced annotation pipelines. This resource addresses a critical gap in abaca genomics and supports future studies on fiber-trait biology, stress resilience, and breeding innovation.

## 2 Results

### 2.1 Genome assembly and characteristics

Abaca (*Musa textilis* cv. Inosa) was sequenced using the PacBio Sequel II Single-Molecule Real-Time (SMRT) platform and assembled using Hifiasm, a haplotype-resolved *de novo* assembler optimized for PacBio HiFi reads. High-fidelity (HiFi) reads generated through circular consensus sequencing (CCS) provided long and highly accurate sequences suitable for producing a highly contiguous genome assembly.

GenomeScope v2 estimated the genome size of Inosa to be approximately 591–616 Mb, depending on k-mer size. The *de novo* assembly spans 612.5 Mb, consistent with k-mer estimates and falling within the range reported for other *M. textilis* genomes, including Abuab (~650.9 Mb scaffolded assembly), the chromosome-level reference assembly (531.05 Mb; GCA_964270295.1), and the genome reported by Zhou et al. (2024; 613.06 Mb). The Inosa genome size is also larger than those of other *Musa* species such as *M. acuminata* ‘Banksii’ (464 Mb), *M. balbisiana* (492 Mb), *M. ornata* (477.18 Mb), and *M. velutina* (496.23 Mb), suggesting potential lineage-specific genome size variation within *M. textilis.*

Assembly statistics (**Table 1**) highlight the high intrinsic contiguity of the Inosa genome. The *de novo* assembly comprises 388 contigs with a contig N50 of 9.02 Mb, substantially higher than the contiguity of the Abuab assembly (contig N50 = 44 kb). Notably, the contig N50 of Inosa also surpasses those of other chromosome-level M. *textilis* assemblies, including GCA_964270295.1 (4.15 Mb) and Zhou (2.92 Mb), indicating high intrinsic contiguity. The GC content (39.18%) is consistent with previously reported abaca genomes (~38.4–39.3%), supporting the absence of compositional bias.

**Table 1:** Assembly statistics of the whole-genome assemblies of abaca with their country of origin.

Assembly	Genome size (Mb)	No. of sequences (contigs/scaffolds)	Scaffold N50 (Mb)	Contig N50 (Mb)	GC (%)	Ns (%)	Assembly level	BUSCO (%) *	Reference
Inosa (de novo)	612.50	388 (contigs)	NA	9.02	39.18	0	Contig	98.90	This study
Inosa (scaffolded)	612.52	200 (scaffolds)	61.77	9.02	39.18	0	Chromosome-scale	98.90	This study
Abuab	650.87	48557 (scaffolds)	0.047	0.04	39.07	0.1	Draft	83.10	Galvez et al., 2021
MTEXT_v1 (GCA_964270295.1)	531.05	10 (scaffolds)	52.06	4.15	38.44	0	Chromosome-scale	96.90	NCBI Assembly GCA_964270295.1 (2024)
Zhou	613.06	83 (scaffolds)	59.31	2.92	39.29	0	Chromosome-scale	97.60	Zhou et al., 2024

*embryophyta_odb12.

Reference-guided scaffolding using RagTag further improved genome organization by anchoring approximately 96% of the assembly into 10 chromosome-scale pseudomolecules, consistent with the expected haploid chromosome number. This resulted in a scaffold N50 of 61.77 Mb, comparable to other chromosome-scale assemblies such as GCA_964270295.1 (52.06 Mb) and Zhou (59.31 Mb). The final assembly comprises 200 scaffolds, including both chromosome-scale sequences and a small proportion (~4%) of unplaced scaffolds. The scaffolded assembly retained the high contig-level continuity (contig N50 = 9.02 Mb), indicating that long-range structure was improved without compromising the underlying assembly quality (**Supplementary Table 1**).

The BUSCO completeness score for Inosa reached 98.9% complete, outperforming the Abuab draft genome (83.1%) and comparable to high-quality chromosome-scale assemblies such as GCA_964270295.1 (96.9%) and Zhou (97.6%). Read mapping further supported assembly quality, with 97.06% of reads aligning to the genome, indicating high completeness and base-level accuracy.

**Figure 1:**
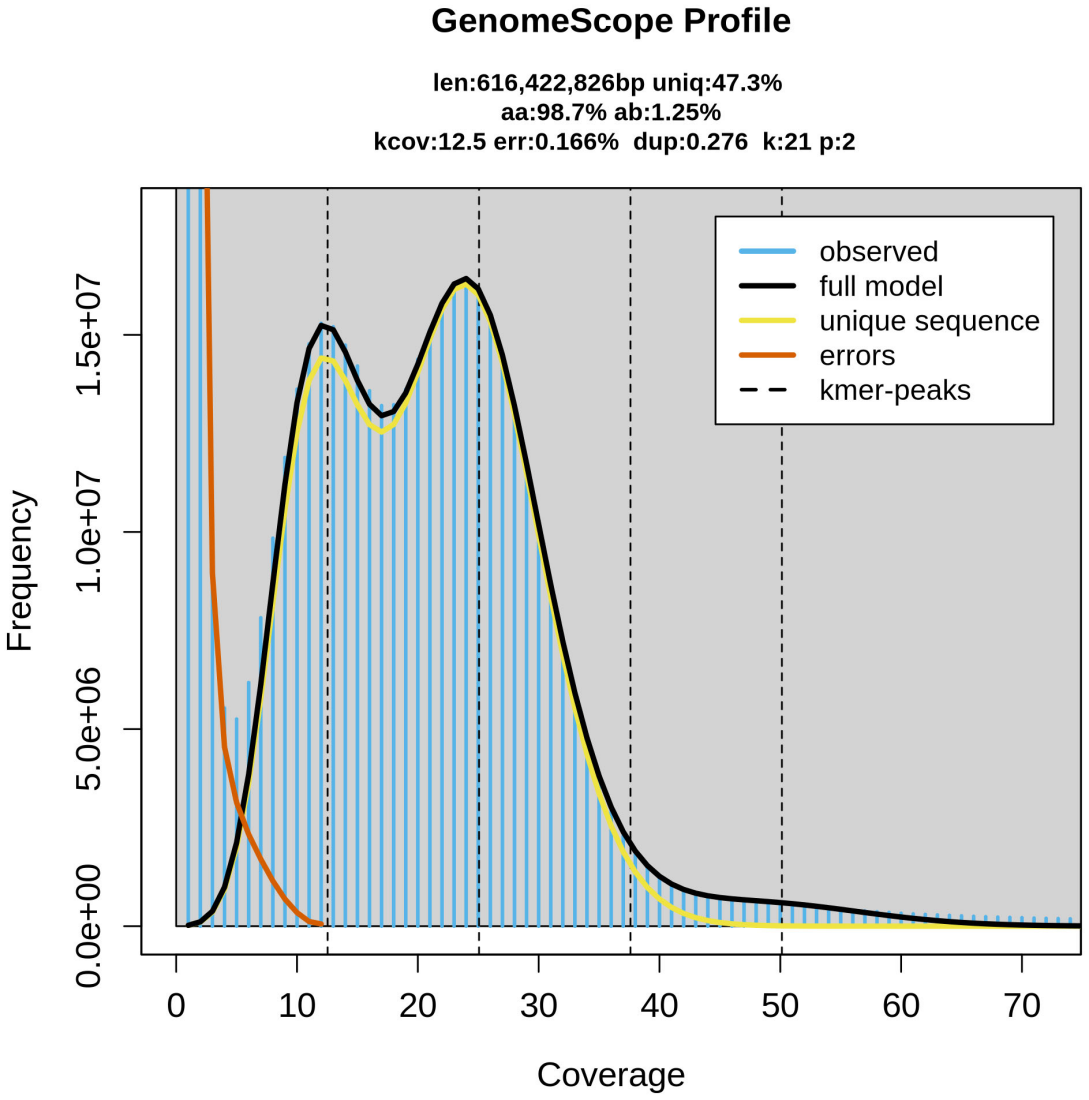
High-quality sequencing and genome profile of *musa textilis* cv. inosa. The GenomeScope profile summarizes k-mer frequency distributions and model fits to estimate genome characteristics. The clear bimodal distribution of k-mer coverage (with blue bars showing the observed counts) is in line with diploidy and backs up a genome size estimate of about 616 Mb (k = 21). The profile further indicates a moderately heterozygous genome (~1.25%), with a low estimated sequencing error rate (~0.17%), consistent with high-quality PacBio HiFi data.

K-mer analysis (**Figure 1**) indicates that the *M. textilis* cv. Inosa genome is diploid, as evidenced by a bimodal k-mer distribution with peaks at approximately ~12× and ~24× coverage, corresponding to heterozygous and homozygous k-mers, respectively. The genome exhibits moderate heterozygosity (~0.8–1.25%), consistent with previous reports for abaca. The GenomeScope model showed a strong fit (>98%) and a low estimated sequencing error rate (~0.11–0.17%), further supporting the high quality of the HiFi-based assembly.

Overall, the Inosa genome assembly exhibits both high intrinsic contiguity at the contig level and chromosome-scale organization after scaffolding, placing it among the most complete and contiguous *Musa textilis* genome resources currently available. The combination of long-read sequencing and reference-guided scaffolding provides a robust framework for downstream analyses, including gene annotation, structural variation, and comparative genomics.

### 2.2 Transposable elements

Transposable element (TE) analysis showed that 59.18% of the Inosa genome (about 297–300 Mb) is made up of TEs consistent with previously reported *Musa* genomes. Long terminal repeat (LTR) retrotransposons were the most common type, with Copia (30.46%) and Ty3-retrotransposons (11.36%) being the most common (**Table 2**). DNA transposons were also detected, including Mutator (2.59%) and CACTA (0.89%). The expansion of Copia elements in Inosa compared to Abuab highlights notable differences between the two assemblies, underscoring the role of TEs in shaping genome size and structure. Beyond contributing to genome expansion, TEs are known to influence gene regulation and genome evolution: they can introduce novel promoters, enhancers, or small RNAs that influence gene expression, and their mobilization has been associated with stress adaptation in *Musa* (Rouard et al., 2018).

**Table 2:** Transposable Elements of three abaca genomes.

Class	Inosa		Abuab		Abaca	
	(this study)		(Galvez et al., 2021)		(Zhou et al., 2024)	
	Count	% masked	Count	% masked	Count	% masked
LTR						
Copia	232,537	30.5	171,310	21.8	189,683	33.8
Ty3-retrotransposons	105,921	11.4	49,839	7.4	86,949	16.4
Unknown	91,195	6.8		19.0	45,034	5.9
TIR						
CACTA	18,358	0.9	21,359	0.7	6,721	0.2
Mutator	44,110	2.6	41,992	1.9	12,422	0.5
PIF						
Harbinger	1,062	0.1	4,880	3.8	764	0.0
Tc1						
Mariner	2,538	0.1	13,409	0.1	919	0.0
hAT	14,116	0.9	17,768	0.9	6,825	0.4
Polinton	122	0.0	-	0.0	-	0.0
Non-LTR						
LINE	723	0.0	4,556	0.9	1,845	0.2
SINE				0.0	26	0.0
Non-TIR						
Helitron	19,733	1.4	10,563	1.0	147	0.0
Repeat Region	78,506	4.6	92,238	1.5		
Total Interspersed	608,921	59.2	497,082	59.4	372,997	58.4

Among the different abaca annotations, only Inosa contained Polintons (also known as Mavericks). We have identified 122 polinton elements in the Inosa genome. These large DNA transposons are thought to represent evolutionary intermediates between transposons and DNA viruses (Krupovic et al., 2014). The presence of polintons is associated with horizontal gene transfer and genome plasticity in other systems (Widen et al., 2023). Another important family is Tc1/Mariner, a widespread group of cut-and-paste DNA transposons that use a DDE transposase. Tc1/Mariner elements are known for their regulatory impact and contribution to genetic diversity, but they have been associated with genome insatability in some systems (Feschotte and Pritham, 2007). Differences in the relative abundance of these DNA transposons between Inosa and Abuab suggest lineage-specific TE dynamics, which may have influenced genome evolution in abaca cultivars.

### 2.3 Structural and functional annotation

Gene prediction in the Inosa genome was performed using RNA-seq datasets from both Inosa and Abuab (Ereful et al., 2022; 2026), combined with protein homology evidence from Viridiplantae orthologs in OrthoDB v11. Unlike the Abuab genome, which employed MAKER for annotation (Galvez et al., 2021), Inosa utilized BRAKER2 (Brůna et al., 2021), an automated pipeline that integrates RNA-seq and protein homology evidence for evidence-guided gene prediction. BRAKER has a number of advantages over MAKER, such as less need for manual curation of training sets, better sensitivity for finding weakly expressed or lineage-specific genes, and better scalability for TE-rich plant genomes (Hoff et al., 2016; Brůna et al., 2021). Using BRAKER resulted in a final set of 37,403 non-redundant protein-coding genes (**Supplementary Table 2**), with an average protein length of 402 amino acids, consistent with other *Musa* genomes (Wu et al., 2016).

Functional annotation of the predicted proteome was performed using InterProScan and eggNOG-mapper (**Supplementary Table 4**). A total of 26,215 (70.0%) were assigned to at least one conserved domain or Gene Ontology (GO) term, yielding 1,127 unique GO terms. GO annotations revealed enrichment in protein binding (GO:0005515; 5,354 counts), ATP binding (GO:0005524; 2,213), and membrane-associated components (GO:0016020; 1,879), suggesting enrichment in interaction networks and membrane-localized functions.

Processes related to signaling and regulation were also highly represented, including protein phosphorylation (GO:0006468; 1,617), protein kinase activity (GO:0004672; 1,609), and regulation of transcription (GO:0006355; 1,550). RNA binding (GO:0003723; 980) further indicated extensive post-transcriptional regulation.

Protein domain analysis using InterProScan identified abundant conserved domains, including leucine-rich repeats (PF00560; 1,860 counts), PPR repeat families (PF01535; 1,569 and PF13041; 1,079 counts), and protein kinase domains (PF00069, 1,059 counts), highlighting extensive signaling and post-transcriptional regulatory capacity.

Orthology-based classification using eggNOG-mapper assigned 31,660 proteins (~84.6%) to Clusters of Orthologous Groups (COG) categories. The most important functions are amino acid transport and metabolism (E; 10.20%), lipid transport and metabolism (I; 9.12%), cytoskeleton-related functions (N; 8.89%), RNA processing and modification (A; 8.37%), signal transduction mechanisms (T; 8.14%), and posttranslational modification, protein turnover, and chaperones (O; 8.12%). General function prediction (R; 6.58%) and function unknown (S; 5.91%) categories were also well represented, reflecting conserved yet partially characterized proteins and lineage-specific genes typical of non-model plant genomes.

KEGG pathway analysis revealed strong enrichment for global metabolic pathways (ko01100; 3,258 hits) and secondary metabolite biosynthesis (ko01110; 1,772 hits), indicating a metabolically active genome with substantial capacity for regulatory and stress-responsive processes.

### 2.4 Genes associated with fiber quality

To examine the genetic foundation of fiber development, genes linked to cell wall biosynthesis and modification were identified through functional annotations. A total of 348 candidate genes linked to fiber biosynthesis and cell wall development provide candidate genes associated with fiber development. These genes include cellulose synthases (34) with a conserved cellulose synthase domain (PF03552), xyloglucan endotransglycosylases (162) (XTH/XET) that help change the shape of the cell wall, and lignin-associated enzymes (152).

Lignin-associated enzymes, such as peroxidases (EC 1.11.1.7), were also found and linked to the phenylpropanoid biosynthesis pathway (ko00940), which suggests involvement in lignin biosynthesis pathways. Genes involved in carbohydrate metabolism and cell wall polysaccharide modification, such as glycoside hydrolases, were also observed.

Transcription factor analysis identified a variety of regulatory genes that may play a role in fiber development, such as MYB (714), AP2/ERF (292), bHLH (257), NAC (191), WRKY (184), bZIP (130), MADS (89), and C2H2 (51) families. These transcription factors represent key regulatory components associated with gene expression and developmental processes.

### 2.5 Comparative genomics and synteny

OrthoFinder analysis across six species (*A. thaliana, M. balbisiana, M. textilis* cv. Inosa, *M. textilis* cv. Abuab, *M. textilis* GCA_964270295.1, and *M. textilis* (Zhou et al., 2024)) clustered 369,302 protein-coding genes into 29,124 orthogroups. In total, 93.3% of genes (344,666) were assigned to orthogroups, while 6.7% (24,636) remained unassigned.

**Figure 2:**
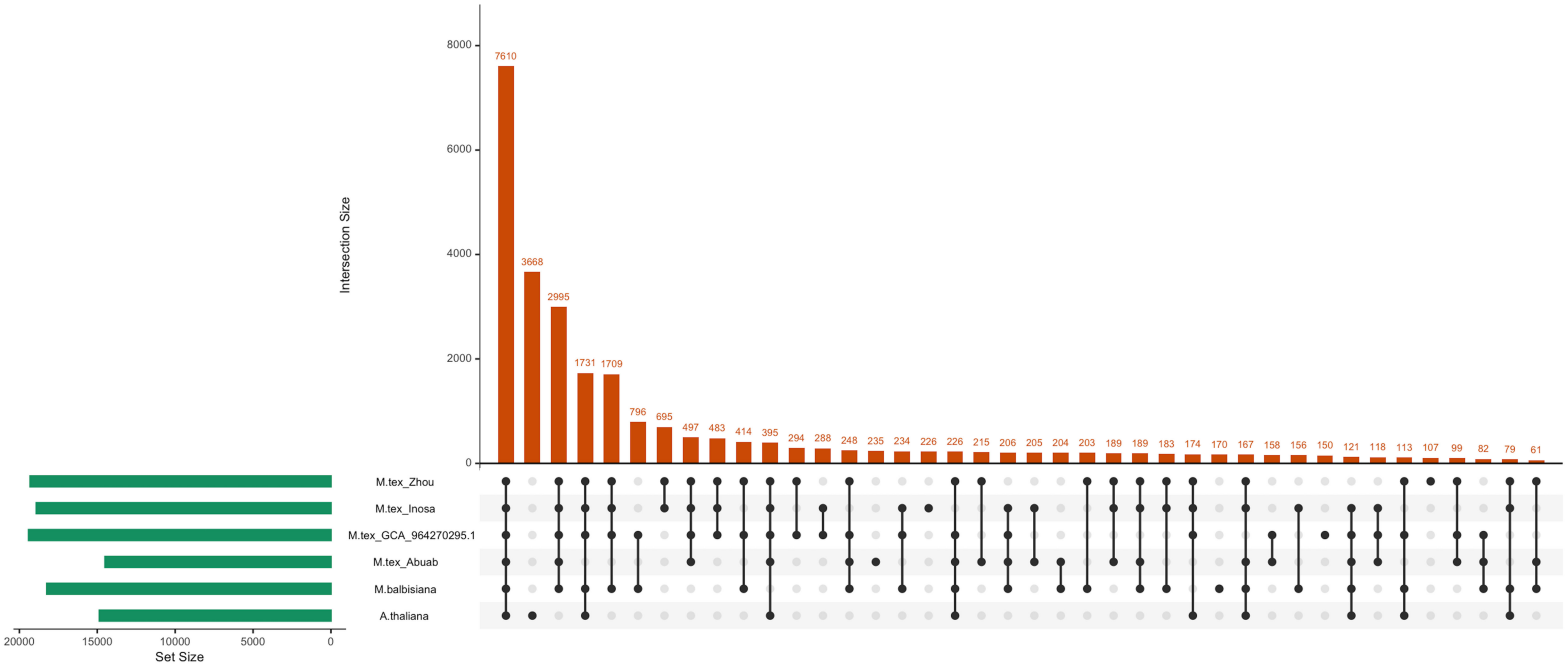
Orthologous group distribution highlights abaca-specific gene innovations. UpSet plot showing the intersection of orthologous groups across six plant species, including four *Musa textilis* assemblies. The plot shows that all six genomes share a large set of core orthogroups (7,610). The visualization highlights the 497 orthogroups unique to the *M. textilis* lineage, representing candidate genes for abaca’s unique fiber traits and other specialized functions, as well as 226 Inosa-specific orthogroups that offer targets for molecular breeding.

**Figure 2** shows that 7,610 orthogroups are shared by all six assemblies (*Arabidopsis* plus the five *Musa* genomes), representing core gene families that have been conserved since their common ancestor. The next largest intersections are 3,668 orthogroups found only in *Arabidopsis* and 2,995 orthogroups found only in *Musa*. The *Arabidopsis*-only orthogroups may represent dicot-specific genes, while the *Musa*-only groups may correspond to monocot-specific gene families, including functions that may have been adapted for the banana and abaca lineages.

The UpSet analysis reveals that among the *Musa* species, *M. textilis* GCA_964270295.1 harbors the highest number of species-specific orthogroups, whereas Abuab contributes the fewest. Notably, the four *M. textilis* assemblies (Abuab, GCA_964270295.1, Inosa, and Zhou) collectively share 497 orthogroups that are unique to abaca and absent in *A. thaliana* and *M. balbisiana*. These abaca-specific gene families may represent lineage-specific innovations that distinguish abaca from other *Musa* species. Their presence suggests potential association with abaca’s unique fiber traits, such as high tensile strength, durability, and saltwater resistance. Beyond fiber production, these orthogroups may also contribute to stress adaptation and ecological specialization. Identifying and characterizing these gene families provides valuable targets for functional genomics and offers candidate genes for breeding programs aimed at improving fiber quality and resilience in abaca.

These lineage- and variety-specific orthogroups likely underlie functional diversification and ecological adaptation, and in the case of *M. textilis*, may represent valuable gene pools for breeding improved abaca varieties.

**Figure 3:**
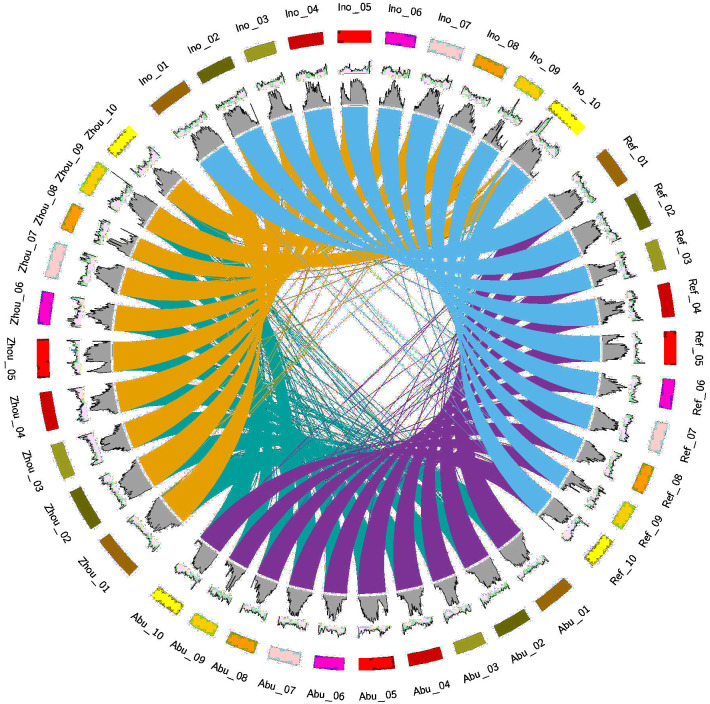
Macrosynteny analysis confirms superior contiguity of the inosa genome. Circos plot illustrating genome-wide synteny between *Musa textilis* cv. Inosa (Ino) and Abuab (Abu) in comparison with a curated Reference Genome (Ref) and the Zhou et al. (2024) assembly. The plot demonstrates strong macrosyntenic conservation across the four genomes (colored ribbons), with Inosa exhibiting notably clean and orderly syntenic blocks against both the Reference and Zhou assemblies. In contrast, the Abuab assembly shows significant crossing ribbons that suggest a higher degree of structural variation, validating the improved contiguity and assembly quality achieved for the Inosa genome.

The reference genome (GCA_964270295) and the genome assembly of Zhou et al. (2024) were compared to the Inosa and Abuab genome assemblies (**Figure 3**). The colored ribbons that connect the chromosomes or scaffolds show conserved syntenic blocks. The color continuity across the four genomes shows macrosyntenic conservation, which means that the order of the genes has been kept mostly the same. Both Inosa and Abuab assemblies exhibit clean synteny with the reference genome. When compared to the assembly of Zhou et al. (2024), however, the Inosa genome is exhibits fewer structural rearrangements than the Abuab, wherein there are significant crossing ribbons (yellow) that highlight potential inversions, translocations, and assembly-specific discrepancies.

## 3 Discussion

The Inosa genome provides an improved genetic resource in abaca, providing a highly contiguous and near-complete assembly of *Musa textilis*. The *de novo* assembly achieved a contig N50 of 9.02 Mb, representing an approximately 200-fold increase over the Abuab assembly (44 kb), and a high BUSCO completeness of 98.9%. Importantly, this high contiguity is intrinsic to the assembly and not solely a result of scaffolding, underscoring the effectiveness of long-read HiFi sequencing. Subsequent reference-guided scaffolding further organized the genome into 10 chromosome-scale pseudomolecules with a scaffold N50 of 61.77 Mb, placing the Inosa assembly at a level comparable to existing chromosome-scale *Musa* references such as GCA_964270295.1 and Zhou et al. (2024).

This genome contributes to the growing body of work on fiber crops, including cotton (*Gossypium*), flax (*Linum*), and ramie (*Boehmeria nivea*), thereby advancing the broader field of fiber plant comparative genomics and adaptation (Yuan et al., 2021; Xu et al., 2023). It provides useful information about the unique gene content and architecture of *Musa textilis*, which remains underrepresented in global pan-genomic studies. The presence of polintons in the Inosa genome can be used to elucidate horizontal gene transfer and its evolutionary implications in pest-host interactions. The Inosa genome contributes to the understanding of how the *Musa* genome evolved in such a complicated way, determine the genetic basis of complex traits (like fiber biosynthesis and disease resistance), and look at intraspecific diversity for pan-genome analyses (Wang et al., 2023). This dataset enhances recent multi-genome assemblies in *Musa* and bolsters evolutionary hypotheses regarding clade divergence, hybridization, and polyploidy (Christelová et al., 2022).

The availability of 37403 high-confidence protein sequences, 348 of which are linked to fiber synthesis and quality, provides a strong foundation for downstream applications. This assembly facilitates accurate localization of genetic variants associated with essential agronomic traits, including fiber strength and yield, as evidenced by studies in banana and rice (Sardos et al., 2022; Yano et al., 2016).

Domain- and annotation-based filtering listed 348 genes associated with fiber synthesis and quality, giving insight into the molecular basis of the superior fiber properties of abaca. These genes include components involved in cellulose biosynthesis, cell wall remodeling, and lignin formation, indicating coordinated regulation of primary and secondary cell wall development. The presence of cellulose synthase candidates and xyloglucan endotransglycosylases (XTH/XET) is consistent with roles in cell wall assembly and restructuring required for fiber elongation, while lignin-associated enzymes, particularly peroxidases in the phenylpropanoid pathway, support secondary cell wall thickening that enhances fiber rigidity and tensile strength. In addition, the abundance of transcription factors, including MYB, NAC, WRKY, and AP2/ERF families, suggests the presence of regulatory networks, with MYB and NAC acting as key regulators of cell wall biosynthesis and WRKY and AP2/ERF integrating developmental and stress-related signals (Zhong and Ye, 2015). Together, these findings support a hierarchical regulatory framework linking gene expression to fiber quality in *Musa textilis.* (Ereful et al., 2022).

The genome is rich in genes and has good annotations, which makes it easier to find trait-associated markers for marker-assisted selection (MAS). The detection of 226 Inosa-specific orthogroups using OrthoFinder highlights potential targets for molecular breeding and genetic improvement (Emms and Kelly, 2019). Furthermore, the annotation of key regulatory genes, including MYB and NAC transcription factors, as well as cellulose synthase genes, provides candidates for investigating the molecular basis of fiber development.

The resource is also suitable for genome editing studies. Inosa is a viable candidate for CRISPR/Cas9-based trait enhancement because it has well-known transcription factors and biosynthetic enzymes (Liu et al., 2021; Jaganathan et al., 2018). These genes may serve as targets for future genome editing studies focused in improving lignin composition, enhancing disease resistance, or optimizing cell wall structure for industrial fiber applications.

Importantly, the Inosa assembly addresses key limitations of previous *Musa textilis* genomic resources. The Abuab genome, while foundational, is highly fragmented and limits downstream analyses. In contrast, the Inosa genome combines high intrinsic contig-level continuity with chromosome-scale scaffolding, resulting in a more complete and structurally coherent reference. This dual advantage facilitates improved gene annotation, structural variation analysis, and pan-genome construction.

Together, these results establish the Inosa genome as a high-quality reference for *Musa textilis*, with direct applications in functional genomics, molecular breeding, and evolutionary studies. The integration of long-read sequencing and reference-guided scaffolding provides a scalable framework for future genomic studies in abaca and other fiber crops.

## 4 Conclusion

This study presents a high-quality genome assembly of *M. textilis* var. Inosa, characterized by high completeness, contiguity, and annotation compared to existing abaca genomes. The identification of extensive repeat content, including unique Polinton elements, and a comprehensive catalog of fiber biosynthesis genes provides a valuable genomic resource for future research on the genetic diversity, fiber quality, and evolutionary adaptations of abaca. This resource may support molecular breeding and biotechnological applications for this important fiber crop.

## 5 Materials and methods

### 5.1 Plant material and nucleic acid extraction

Young leaf tissues of *Musa textilis* cv. Inosa were collected from the germplasm field at the Institute of Plant Breeding, University of the Philippines, Los Baños (UPLB). Samples were flash-frozen in liquid nitrogen and stored at −80 °C until processing. High-molecular-weight genomic DNA was extracted from the flag leaf using a modified cetyltrimethylammonium bromide (CTAB) method optimized for fibrous monocots (Inglis et al., 2018). DNA quality and quantity were assessed using a BioTek Epoch spectrophotometer, Qubit fluorometer (Thermo Fisher Scientific), and 1.5% agarose gel electrophoresis.

### 5.2 DNA sequencing and genome assembly

The *Musa textilis* cv. Inosa genome was sequenced using the PacBio Sequel II platform with HiFi circular consensus sequencing (CCS) chemistry. A total of ~15.9 Gb of HiFi reads were generated, with a read N50 of approximately 15.5 kb, corresponding to an estimated genome coverage of ~27.5× based on the final assembly size. CCS reads were filtered for adapter contamination using HiFiAdapterFilt (Sim et al., 2022).

K-mer counting was performed using Meryl (v1.3) with a k-mer size of 21. Genome size, heterozygosity, and repeat content were estimated using GenomeScope v2.0 (Ranallo-Benavidez et al., 2020) under default parameters.

Filtered HiFi reads were assembled *de novo* using Hifiasm (v0.24.0; Cheng et al., 2022) with default parameters, producing a primary contig assembly. The resulting *de novo* assembly comprised 388 contigs with a total size of ~612.5 Mb and a contig N50 of ~9.02 Mb.

To improve long-range genome organization, reference-guided scaffolding was performed using RagTag (v2.1.0; Alonge et al., 2022) against a chromosome-level *Musa textilis* reference genome (NCBI Assembly GCA_964270295.1). This step generated 10 chromosome-scale pseudomolecules and produced a final assembly consisting of 200 scaffolds, with approximately 96% of the genome anchored to chromosomes and the remaining sequences retained as unplaced scaffolds.

Assembly contiguity and statistics were evaluated using QUAST (v5.3.0; Mikheenko et al., 2018). Genome completeness was assessed using BUSCO (v6.0.0; Tegenfeldt et al., 2025) against the embryophyta_odb12 lineage dataset, reporting the proportion of complete (single-copy and duplicated), fragmented, and missing orthologs.

### 5.3 Gene prediction, structural, and functional annotation

Repeats were predicted using RepeatModeler v. 2.0.7 (Smit and Hubley, 2015) and masked using RepeatMasker v. 4.2.1 (Smit et al., 2015). The masked genome was used for gene prediction and annotation using the BRAKER2 workflow (Brůna et al., 2021). This fully automated pipeline used RNA-Seq data from *Musa textilis* var. Abuab (Accession: PRJEB47952) (Ereful et al., 2026) and a curated Viridiplantae protein from OrthoDB v.11 (Kuznetsov et al., 2023) were used to train and predict highly reliable genes with GeneMark-EP (Brůna et al., 2020; Lomsadze, 2005) and AUGUSTUS (Stanke et al., 2006; Stanke et al., 2008; Lomsadze et al., 2014). Gene prediction using BRAKER2 followed by length filtering, coding potential refinement with TransDecoder v. 5.5.0 (Brian & Papanicolaou, n.d.), and redundancy reduction using CD-HIT at 95% identity threshold (Fu et al., 2012). Structural and functional annotation was achieved using Interproscan v.5.71-102.0 (Jones et al., 2014); BLASTp v. 2.14.1+ (Camacho et al., 2009) using the non-redundant (nr) database; and eggNOG mapper v5.0 (Huerta-Cepas et al., 2019).

### 5.4 Orthology and comparative genomics

Protein sequences from *Musa balbisiana*, *Arabidopsis thaliana*, *M. textilis* cv. Abuab, *M. textilis* GCA_964270295.1, *M. textilis* (Zhou et al., 2024) were retrieved from NCBI. Orthologous gene clusters were inferred using OrthoFinder v2.5.5 (Emms and Kelly, 2019) with default parameters. Shared and species-specific orthogroups were visualized using the UpSet diagram generated in R using ggplot2 (Wickham, 2016). Syntenic relationships were explored using Circos (Galaxy Version 0.69.8+galaxy9) (Krzywinski et al., 2009; Rasche and Hiltemann, 2020), where the Inosa and the Abuab (Galvez et al., 2021) assemblies were aligned to the *M. textilis* reference genome and the assembly of Zhou et al., 2024. Synteny plots were used to assess macrosyntenic conservation and identify potential structural variations, such as inversions and translocations.

### 5.5 Transposable element annotation

Repetitive elements were identified using RepeatMasker and Extensive *de novo* TE Annotator (EDTA) v.2.2.2 (Ou et al., 2019). TEs were classified into LTRs, non-LTRs, TIRs, and other structural classes. A comparison of repeat classes among *Musa* genomes was conducted to assess genome dynamics. Novel polinton elements were carefully curated and compared with published TE databases.

## Data Availability

The datasets presented in this study can be found in online repositories. The names of the repository/repositories and accession number(s) can be found below: https://www.ncbi.nlm.nih.gov/genbank/, PRJNA1209681.
