# Supplementary material for: High-quality draft genome assembly and functional annotation of Musa textilis cv. Inosa
Source: Front Plant Sci. 2026 Jul 7;17:1866360. doi: 10.3389/fpls.2026.1866360 (PMC13386271; doi:10.3389/fpls.2026.1866360)
Supplement: Supplementary file 1 [file Table1.docx]

Supplementary Table 1. Assembly continuity and chromosome anchoring statistics of the Musa textilis cv. Inosa genome. Assembly metrics were calculated using QUAST and chromosome-scale scaffolding statistics were generated using RagTag.

| **Category** | **Metric** | **Value** |
| --- | --- | --- |
| **Assembly statistics** | Total assembly size (bp) | 612,502,812 |
|  | Number of contigs | 388 |
|  | Largest contig (bp) | 25,345,336 |
|  | GC content (%) | 39.18 |
|  | Contig N50 (bp) | 9,017,123 |
|  | Contig N90 (bp) | 1,607,447 |
|  | auN (bp) | 9,726,792 |
|  | L50 | 23 |
|  | L90 | 83 |
|  | Ns per 100 kbp | 0 |
| **Chromosome anchoring (RagTag)** | Placed sequences | 200 |
|  | Anchored sequence length (bp) | 588,080,460 |
|  | Anchored genome fraction (%) | 96 |
|  | Unplaced sequences | 188 |
|  | Unplaced sequence length (bp) | 24,422,352 |
|  | Unplaced genome fraction (%) | 4 |
|  | Gap sequences introduced | 188 |
|  | Gap bases introduced (bp) | 18,800 |
|  | BUSCO completeness (%) | 98.90 |

Supplementary Table 2. Structural characteristics of the final isoform-filtered gene annotation set generated for Musa textilis cv. Inosa.

| **Category** | **Metric** | **Value** |
| --- | --- | --- |
| **Gene prediction** | Total predicted protein-coding genes | 55,659 |
| **Protein prediction** | Total predicted proteins | 59,130 |
|  | Total predicted non-redundant protein | 37,403 |
|  | BUSCO completeness of proteins (%) | 85.4 |
| **Gene structure** | Single-exon genes | 12,169 (21.9%) |
|  | Multi-exon genes | 43,490 (78.1%) |
|  | Mean gene length (bp) | 3,608 |
|  | Mean exons per gene | 4.9 |
|  | Mean introns per gene | 3.9 |
|  | Mean exon length (bp) | 206 |
|  | Mean intron length (bp) | 667 |
|  | Longest gene (bp) | 96,051 |

Table 3. Functional annotation coverage and classification of predicted genes based on InterProScan and eggNOG analyses.

| **Source** | **Metric** | **Count** | **Percent (%)** |
| --- | --- | --- | --- |
| **InterProScan** | Genes with annotation hits | 26,215 | 70.1 |
|  | Genes with InterPro IDs | 25,522 | 68.2 |
|  | Genes with GO annotations | 15,221 | 40.7 |
| **eggNOG** | Genes with functional descriptions | 29,610 | 79.2 |
|  | Genes assigned GO terms | 15,400 | 41.2 |
|  | Genes assigned KEGG orthologs | 15,237 | 40.7 |
|  | Genes assigned KEGG pathways | 9,418 | 25.2 |
|  | Genes assigned COG categories | 29,610 | 79.2 |
|  | Genes with PFAM domains | 28,416 | 76 |
|  |  |  |  |
